# The Association between Peptide Hormones with Obesity and Insulin Resistance Markers in Lean and Obese Individuals in the United Arab Emirates

**DOI:** 10.3390/nu14061271

**Published:** 2022-03-17

**Authors:** Manal Ali Ahmad, Mirey Karavetian, Carole Ayoub Moubareck, Gabi Wazz, Tarek Mahdy, Koen Venema

**Affiliations:** 1 School of Nutrition and Translational Research in Metabolism (NUTRIM), Faculty of Health, Medicine and Life Sciences, Maastricht University, 6200 MD Maastricht, The Netherlands; manale.aa@hotmail.com; 2 Department of Food and Nutrition, Ryerson University, Toronto, ON M5B 2K3, Canada; mirey.karavetian@ryerson.ca; 3 College of Natural and Health Sciences, Zayed University, Dubai 19282, United Arab Emirates; carole.ayoubmoubareck@zu.ac.ae; 4 Center of Excellence in Bariatric and Metabolic Surgery, Dr. Sulaiman Al Habib Hospital, Dubai 505005, United Arab Emirates; gabiw@drsulaimanalhabib.com; 5 Department of Bariatric Surgery, Sharjah University, Sharjah 27272, United Arab Emirates; tmahdy@yahoo.com; 6 Centre for Healthy Eating & Food Innovation, Faculty of Science & Engineering, Campus Venlo, Maastricht University, 5928 SZ Venlo, The Netherlands

**Keywords:** obesity, brain–gut axis, insulin resistance, glucagon-like peptide-1, glucagon-like peptide-2, insulin, leptin, cholecystokinin, peptide yy, ghrelin

## Abstract

Peptide hormones play a crucial role in body weight and glucose homeostasis. In this study, we aimed to explore this association and recruited 43 obese and 31 age- and sex-matched lean participants. We assessed their body mass index (BMI), waist circumference (WC), waist-to-height ratio (WtHR), percentage body fat (PBF), fasting blood levels of peptide hormones (GLP-1, GLP-2, insulin, leptin, ghrelin, CCK, and PYY), fasting blood sugar (FBS), and Homeostatic Model Assessment of Insulin Resistance (HOMA-IR). We tested the associations between peptide hormones and markers of obesity and insulin resistance (IR) by using the Independent-Samples *t*-test and Mann-Whitney U test, partial correlation, and logistic regression. FBS, insulin, HOMA-IR, GLP-1, GLP-2, and leptin were significantly higher in the obese group; ghrelin and CCK were significantly higher in lean participants, and no difference was seen for PYY. Controlling for BMI, GLP-1 was positively correlated with WtHR, while ghrelin was inversely correlated with WtHR. GLP-1 was correlated with HOMA-IR. GLP-1 was associated with obesity and IR markers in the regression model. Our results show that obese and lean adults display significant differences in plasma peptide hormone levels. GLP-1 levels were independently associated with markers of obesity and IR. Restoring the appetite hormone balance in obesity may represent a potential therapeutic target.

## 1. Introduction

Obesity is reaching unprecedented epidemic levels. Globally, in 2016, 39% and 13% of adults were estimated as overweight and obese, respectively [1]. Extending from its simplistic description as an imbalance between energy intake and expenditure, obesity ensues from multifaceted interactions between genetic, environmental, psychological, lifestyle, and dietary factors, making it a complex disease to understand and address [2]. Specifically, a growing body of evidence supports the role of the gut–brain axis in the pathogenesis of obesity [3]. On the one hand, the brain signals to the gut via efferent vagal and neuroendocrine pathways [4], and on the other hand, peptide hormones act as signaling molecules [5], triggering autonomic reflexes to regulate appetite, energy, and glucose homeostasis [6]. These peptides are divided into short-term effectors, such as glucagon-like peptides (GLP)-1 and -2, cholecystokinin (CCK), peptide YY (PPY), and ghrelin, and long-term effectors, including insulin, leptin, and adipokines [7]. Significant alterations in the expression and secretion of peptide hormones may disrupt intermediary metabolism, ultimately manifesting in increased body weight and insulin resistance (IR), leading to diabetes, metabolic syndrome, cardiovascular disease, or dyslipidemia [8]. In obesity, neural, gut- and adipose-tissue-derived hormone signaling are altered [9]. For instance, compared with normal-weighted individuals, people with obesity have reduced fasting ghrelin levels which could be a counter-regulatory mechanism to excess body weight and reduced post–meal suppression [10].

Furthermore, individuals with obesity have reduced fasting levels of PYY and GLP-1 and blunted meal-stimulated levels of these anorectic peptides, in addition to an attenuated satiety-promoting effect of CCK [11], leptin resistance, and IR [12,13]. However, it is unclear whether these alterations are a cause or a consequence of obesity [14]. Moreover, microbiota dysbiosis has been associated with impairments in the secretion of many peptide hormones, coupled with increased appetite, enhanced energy harvest from food intake, and fat storage [15]. Apart from bariatric surgery, treatment options for obesity are ineffective in achieving sustainable weight loss [16], making the search for potential alternative treatments a necessity [14]. The effectiveness of a bariatric surgery procedure could partly be lying in its effect on peptide hormones and subsequent regulation of energy homeostasis [16].

Nevertheless, surgical treatment of obesity can never be the sole solution, as it is an invasive, risky, and costly procedure [17]. Consequently, the field of neuroendocrinology, specifically the modulation of peptide hormones, holds promising therapeutic implications [10]. However, the definitive roles of each of these molecules remain not fully understood and are yet to be uncovered [18]. Further research is needed to address knowledge gaps and unresolved issues in the field of peptide hormones and obesity [11]. Understanding underlying pathophysiological links have the potential to allow for more accurate identification of high-risk participants and open the door for new therapeutic implications [19]. This is of utmost importance for communities exhibiting alarming rates of obesity, such as in the United Arab Emirates (UAE), amounting, in 2016, to 31.6% among men and 41.2% among women [20]. Accordingly, this study aimed to assess plasma peptide hormones within the context of obesity and IR among lean and obese adult Emirati participants.

## 2. Materials and Methods

### 2.1. Study Design

This was a cross-sectional comparative study between lean and obese Emirati individuals residing in the UAE. It was part of a pre–post study exploring the effect of a bariatric procedure on gut microbiota, peptide hormones, food intake, and metabolism. The study was registered on ClinicalTrials.gov (ID: NCT04200521). Raw data are available at the Mendeley Data repository (https://data.mendeley.com/datasets/w297t54m98/1, accessed on 7 February 2022) [21].

### 2.2. Ethics Statement

All participants gave their informed consent for inclusion before they participated in the study. The study was conducted in accordance with the Declaration of Helsinki, and the protocol was approved by the Ethics Committee of the Ministry of Health and Prevention (MOH), Dubai Health Care Regulatory Research Ethics Committee (DHCR-REC), and the Zayed University Ethical Committee Board (ZU19_51_F).

### 2.3. Participants and Sample Size

The current study utilized data from a mother study that aimed to assess gut microbiota among lean and obese Emirati participants and changes in microbiota post-bariatric surgery. In the mother study, the primary outcome was the change in the Firmicutes-to-Bacteroidetes ratio. The sample size calculation was performed according to change in Bacteroidetes/Firmicutes between pre- and post-bariatric procedure [22] based on the study by Damms-Machado et al. [23] where the mean (SD) fecal Bacteroidetes-to-Firmicutes ratio changed significantly from 5.9 (2.1) to 10.4 (1.4) in 3 months post-bariatric surgery [23] A sample size of 2 participants was required to achieve an 80% power at a two-sided 5% significance level (Ali Ahmad et al., in preparation). To allow for the high expected dropout rate, the minimum sample size was multiplied by 15. Hence, a total of 30 obese planning to undergo a bariatric procedure, and 30 lean Emirati, matched by age and sex, were targeted. 

Eligibility criteria included stable body weight and free of antibiotic use for the last three months, between 18 and 60 years, residing in the UAE, from both sexes. Consenting participants with a body mass index (BMI) ≥ 35 kg/m^2^ planning to undergo bariatric surgery with a clearance from the bariatric surgeon were assigned to the “obese” study group. Afterward, individuals with a BMI between 18.5 and 24.9 kg/m^2^, matched for age- and sex of the initially recruited obese participants, were assigned to the “lean” study group. Exclusion criteria were as follows: weight loss ≥ 5% in the last three months, pregnancy, alcohol consumption of more than two drinks in a day for men and more than one drink in a day for women [24], or unwilling to provide written informed consent. Obese participants were recruited via a direct approach from two hospitals (one in Dubai and one in Sharjah), whereas lean participants were recruited from the general community in Dubai, using flyer postings and word of mouth. Recruitment was conducted between October 2019 and March 2021.

### 2.4. Outcome Measures and Data Collection 

Inclusion criteria were assessed by using a screening questionnaire addressing general health, weight (loss), medication and supplement use, physical activity, antibiotic usage, alcohol consumption, and comorbidities. Eligible participants were asked to avoid alcohol consumption and any strenuous exercise 24 h before the screening and come for data collection after a 12-h fast. Outcome measures, collected by trained dietitians and research assistants, included the following:

### 2.5. Biochemical Parameters

Peptide hormones: venous blood samples were collected after fasting for 12 h, using a 22-gauge needle, by a licensed nurse, following standard techniques and three vacutainers tubes. One 5 mL sample was drawn into Ethylenediaminetetraacetic acid (EDTA) tube to analyze GLP-1, GLP-2, insulin, leptin, and CCK without adding any stabilizer. Another two 3-mL samples were drawn into EDTA tubes. For the first one, 30 μL of Pefabloc (Aminoethyl-benzenesulfonyl fluoride hydrochloride, i.e., AEBSF (Sigma Aldrich, St. Louis, MO, USA; 100 mg/mL in distilled water) was added to stabilize ghrelin. For the other tube, 30 μL of DPP IV inhibitor (Sigma Aldrich) was added to stabilize PYY. According to the manufacturer’s instructions, hormones were analyzed by using Enzyme-Linked Immunosorbent Assay (ELISA). Kits used were purchased from Diametra Millipore (St. Louis, MO, USA) for GLP-1, GLP-2, PYY, ghrelin, insulin, and leptin; and ABclonal Technology (Wuhan, China) for CCK. 

Fasting blood sugar (FBS) was measured by using a portable Lux Meter Blood Test following the manufacturer’s instructions (Biochemical Systems International, S.p.A; Arezzo, Italy).

Homeostatic Model Assessment of IR (HOMA-IR) was calculated as fasting serum insulin (mU/mL) × FBS (mg/dL)/405 [25]; values higher than 1.8 denoted IR [26].

### 2.6. Anthropometric Measurements

Height and weight were measured to determine BMI. Body weight (Kg) was assessed by using an electronic portable Seca 762 scale (Vogel & Halke, Hamburg, Germany), without shoes, with the participant wearing light clothes. Height (cm) was measured by using a portable stadiometer attached to the Seca weighing scale and measured to the nearest 0.1 cm, without shoes, with the participant stretching to the maximum height. BMI (kg/m^2^) was calculated by dividing kilogram weight by squared height in meters.

Waist circumference (WC) (cm) was measured to the nearest 0.1 cm at the mid-point, halfway between the right iliac crest and the lower costal region [27], using measuring tapes Seca 201 Ergonomic Circumference Measuring Tape. WC values were categorized as elevated according to the cut-off points of ≥94 cm for men and ≥80 cm for women [28].

Waist-to-height ratio (WtHR) was calculated by dividing the WC (cm) by height (cm). Values ≥ 0.5 indicated abdominal obesity [29].

Percent body fat (PBF) was evaluated by using a bioelectrical impedance analyzer (BC-420 MA, Tanita Corporation, Tokyo, Japan). Participants were asked to be well hydrated, to not drink caffeine for 12 h, and to not participate in any excessive physical activity 24 h before the body-composition analysis. Cutoffs of ≥25% and ≥35% were used to define elevated PBF among men and women, respectively [30].

### 2.7. Statistical Analyses 

Statistical analyses were performed by using the Statistical Package for the Social Sciences software version 21.0 (SPSS Inc., Chicago, IL, USA). The normality of the data was tested by using skewness and kurtosis. Data were expressed as means ± standard deviations and frequencies and percentages for continuous and categorical variables, respectively. Differences between the obese and lean participants were examined by using Independent-Samples *t*-test and Mann-Whitney U test for normally distributed and skewed continuous variables, respectively, and Chi-square for categorical variables. Pearson and Spearman’s correlations were used to assess the correlations between peptide hormones (GLP-1, GLP-2, insulin, leptin, CCK, PYY, and ghrelin) and markers of obesity and IR (BMI, WC, WtHR, PBF, insulin, FBS, and HOMA-IR). These analyses were followed by partial correlations, adjusting for BMI, for all variables, except for BMI. A correlation coefficient (*r*) ranging between |0.10| and |0.39|; |0.4| and |0.69|; |0.70| and |0.89|; and |0.90| and |1.00| denoted a weak, moderate, strong, and very strong correlation, respectively [31]. Binary regression analyses were conducted by using the forward method, with categories of weight status, HOMA-IR, WC, WtHR, and PBF being the dependent variables and the peptide hormones being the independent variables. The number of independent variables entered into each model was set at 10% of the group with the outcome of interest. The choice of the independent variables was based on the results of the bivariate analyses (*p* < 0.2) and the pertaining scientific literature. A *p*-value less than 0.05 was considered statistically significant.

## 3. Results

In total, 74 adults were included: 43 obese (16 males, 27 females) and 31 lean (12 males, 19 females). Their characteristics are detailed below in Table 1. There was no between-group significant difference regarding age and sex. Obese participants exhibited significantly higher levels of FBS, insulin, HOMA-IR, GLP-1, GLP-2, and leptin compared with lean participants. In contrast, lean participants showed significantly higher levels of ghrelin and CCK than obese participants, while no between-group differences were noted regarding PYY. As for comorbidities, 2.3% and 11.6% of the obese group had dyslipidemia and hypertension, respectively.

The results of the correlation analyses between peptide hormones and markers of obesity and IR for the whole sample are detailed in Table 2. All peptide hormones were significantly correlated with BMI, whereby GLP-1, GLP-2, insulin, and leptin showed a positive correlation (*r* = 0.426, *p* < 0.001; *r* = 0.403, *p* < 0.001; *r* = 0.61, *p* < 0.001; *r* = 0.801, *p* < 0.001, respectively), whereas CCK and ghrelin showed a negative correlation (*r* = −0.356, *p* = 0.002; *r* = −0.680, *p* < 0.001, respectively). Specifically, GLP-1, GLP-2, insulin and leptin were positively correlated with WtHR (*r* = 0.549, *p* < 0.001; *r* = 0.444, *p* < 0.001; *r* = 0.651, *p* < 0.001; *r* = 0.756, *p* < 0.001, respectively), while CCK and ghrelin were negatively correlated with WtHR (*r* = −0.291, *p* = 0.012; *r* = −0.706, *p* < 0.001). Regarding IR markers, GLP-1, GLP-2, and leptin were positively correlated with HOMA-IR (*r* = 0.738, *p* < 0.001; *r* = 0.368, *p* = 0.001; *r* = 0.588, *p* < 0.001), while ghrelin was negatively correlated with HOMA-IR (*r* = −0.514; *p* < 0.001). PYY was not correlated with any of the assessed markers.

The results of partial correlations adjusting for BMI are presented in Table 3. Leptin, GLP-2, and CCK correlations with obesity markers were annulled when adjusting for BMI. In contrast, GLP-1 remained correlated with WtHR (*r* = 0.312, *p* = 0.039), and with WC among females only (*r* = 0.447, *p* < 0.001). Insulin remained positively correlated with WtHR (*r* = 0.275, *p* = 0.019) and ghrelin remained inversely correlated with WtHR (*r* = −0.302, *p* = 0.019). As for the markers of IR, GLP-1 and GLP-2 were positively correlated with insulin (*r* = 0.612, *p* < 0.001; *r* = 0.236, *p* = 0.046, respectively). Moreover, GLP-1 showed a positive correlation with HOMA-IR (*r* = 0.667, *p* < 0.001). None of the peptide hormones was correlated with FBS. Finally, PYY was not correlated with any of the assessed markers.

The binary logistic regression adjusting for BMI results are presented in Table 4 and show that GLP-1 was positively associated with HOMA-IR (odds ratio (OR) = 1.221, and 95% confidence interval (CI) = 1.079–1.382). No significant associations were noted between leptin nor ghrelin and HOMA-IR. Moreover, GLP-1 was positively associated with WC and WtHR (OR = 1.196, and 95% CI = 1.001–1.430; OR = 1.591, and 95% CI = 1.072–2.363, respectively), but negatively associated with PBF (OR = 0.717, and 95% CI = 0.542–0.949). Finally, leptin and GLP-1 were positively associated with obesity (OR = 1.367, and 95% CI = 1.117–1.672; OR = 1.239, and 95% CI = 1.018–1.508, respectively). 

## 4. Discussion

Obesity and its complications are an ever-increasing public-health challenge. The relationship between gut-derived hormonal dysregulation and obesity is long-known [10]. More specifically, peptide hormones, such as PYY, GLP-1, CCK, insulin, leptin, and ghrelin, are implicated in appetite regulation. Of these, only ghrelin is orexigenic, while the others have anorexigenic properties [11]. The interplay between peptide hormones and cardiometabolic outcomes in Arabs has received little attention in the scientific literature, thus justifying the need for this research. Exploring interventions to reduce obesity—specifically in the UAE—is a national health priority, given the alarmingly high obesity rate [32].

This study reported statistically significant differences in GLP-1, GLP-2, insulin, leptin, ghrelin, and CCK between obese and lean participants. Our results further highlight the dysregulation of peptide hormone secretion profiles with obesity and IR [19,33]. Whether these alterations are consequential or inductive to weight gain remains to be explored [34].

Moreover, we found that GLP-1 predicted obesity status and IR, even after controlling for BMI, whereas leptin predicted obesity and its markers, but after controlling for BMI, this association was annulled. As expected, individuals with obesity had higher fasting insulin levels and HOMA-IR than lean participants [35]. Moreover, 92.5% of obese participants had IR, compared with only 7.5% of lean participants. Interestingly, insulin remained positively correlated with WtHR after controlling for BMI, emphasizing the latter as a promising anthropometric screening parameter indicative of IR [36], being a sensitive, inexpensive, non-invasive, simple-to-assess, and easy-to-calculate tool [37,38]. 

We found that fasting GLP-1 was significantly higher in obese participants, as seen elsewhere [39,40]. Notably, GLP-1 was the only independent gut peptide predictor of obesity markers and IR. The inflammatory cytokine interleukin-6 (IL-6) stimulates GLP-1 secretion from pancreatic α-cells, subsequently promoting insulin secretion, hepatic lipogenesis, and adipogenesis, which might explain the positive association between GLP-1 and IR and adiposity [41]. It is also possible that GLP-1 levels are naturally increased as a homeostatic protective mechanism to counteract energy surplus in obese participants [41]. This suggestion is plausible, as overfeeding was shown to increase fasting levels of GLP-1 [42]. Obesity is also associated with intestinal microbiota dysbiosis, altering GLP-1 secretion [40]. Our findings seem to be aligned with the emerging literature [40,41], whereby fasting GLP-1 may be considered an indicator of cardiometabolic risk. Most importantly, these findings suggest therapeutic implications. For instance, GLP-1, an incretin hormone, regulates glucose homeostasis and suppresses appetite [19], and GLP-1 treatment in controlling blood glucose levels, hunger, and energy consumption has been proven effective in recent studies [43,44]. Although obesity affects postprandial GLP-1 secretion, obese participants are sensitive to systematically administered GLP-1 with reduced hunger, slowed gastric emptying [10], and decreased IR [45]. Moreover, administration of GLP-1 receptor agonists in combination with related gut-derived hormones imparts metabolic advantages beyond those seen with GLP-1 alone [46]. 

Obese participants exhibited significantly higher leptin levels, positively correlating with obesity and IR markers [47]. Since leptin is excreted by the adipose tissue [48], a positive correlation between body fat and leptin was expected [49] and was annulled after controlling for BMI. Moreover, leptin levels decrease with a reduction of body fat [50], indicating enhanced leptin sensitivity [12], which correlates with maintaining the weight loss [51]. The concept of leptin resistance may explain the paradoxically elevated leptin levels in obesity, notably through impaired transportation of leptin through the blood-brain barrier [12] and the decreased hypothalamic leptin receptor expression [12]. Preventing and treating leptin resistance may open new therapeutic strategies [12,50], especially since approaches to enhancing leptin sensitivity in animal models have shown some success; however, these findings are without a clinically relevant application to date [12].

While PYY is known to reduce appetite and energy intake, delay gastric emptying, and promote insulin secretion [15], its relationship with obesity and IR remains controversial [19,52]. In our study, no between-group significant differences were noted. We did not find any association between fasting PYY and obesity nor IR, except for a positive correlation with FBS in obese participants. It is highly plausible that, in contrast to other peptide hormones, obesity is not associated with resistance to PYY, thus explaining the lack of difference in its fasting concentrations between lean and obese individuals [53,54]. Furthermore, PYY levels are affected by numerous factors beyond weight. For example, reduced concentrations in people with a high BMI might be related to consuming a low-fiber diet rather than fat stores or IR [55]. Targeted studies examining adiposity and dietary intake are required to establish the underlying causality.

In our study, fasting CCK was significantly higher in lean participants. Moreover, CCK was negatively correlated with all obesity markers, which was annulled after controlling for BMI. CCK is known to reduce energy intake and promote insulin secretion [56]. Whether obesity affects CCK secretion is controversial [19] and could be due to methodological problems in measuring CCK [57]. CCK-resistance in obesity is evident, making obese individuals less sensitive to CCK’s satiety effect [58]. The biological action profile of CCK has strong parallels with those of GLP-1 [56], and a combined activation of their receptors induces body-weight reduction and blood-glucose control [59].

Furthermore, CCK and leptin synergistically inhibit caloric intake [56]. The co-administration of CCK with GLP-1 and leptin substantially enhances metabolic benefits under conditions of obesity and diabetes [56]. CCK combinatorial therapeutics may have yet unexploited therapeutic potential. 

Finally, we found that fasting ghrelin was significantly lower in obese participants, and after controlling for BMI, it was negatively correlated only with WtHR [3]. Fasting ghrelin decreases with obesity and increases by diet-induced weight loss [19]. The underlying mechanisms have not been elucidated, but circulating plasma ghrelin was reported to decrease significantly after food intake [11]. Seemingly, increased caloric intake suppresses plasma ghrelin levels, and reduced ghrelin may be compensatory rather than causal, being a physiological adaptation to the positive caloric balance and excess body weight [10]. In human primary funding cultures, obesity decreased ghrelin production at the cellular protein level, leading to a reduced secretion in the cell supernatant [10].

Moreover, downregulation of ghrelin secretion may result from high IR [60]. Ghrelin administration increases food intake, lowers energy expenditure, and upregulates gene expression of fat-storage-promoting enzymes in white adipose tissue [61]. In contrast, neutralization of bioavailable ghrelin and administration of growth hormone secretagogue receptor or ghrelin-O-acyltransferase antagonists to mice fed a high-fat diet lower body weight and food intake [10].

### Strengths and Limitations

To our knowledge, this is the first study providing information regarding seven peptide hormones in adults Emiratis and exploring the associations between these hormones and validated markers of obesity and IR. Our findings present important implications for clinical practice and future diagnostic and interventional studies, especially that combinatorial therapies of hormones involved in energy regulation act through various pathways and avoid compensatory mechanisms [12]. Moreover, we measured total GLP-1, providing better information about the secretion of the hormone and its effects, compared with measurements of intact (or active) GLP-1 [62]. However, this analysis had several limitations. First, it focused only on fasting peptide hormone concentrations, varying in food intake and macronutrient composition. Exploring postprandial concentrations and their relationship with obesity and IR could provide additional insights into unanswered questions in this study. Moreover, our sample consisted of Emiratis aged 18–60 years. Future studies are needed to uncover whether the relationships identified here hold other age ranges and ethnic groups. Finally, our results are hindered by the relatively small sample size and the study’s cross-sectional nature. Therefore, prospective studies should be carried out with powered samples to validate our findings and uncover potential cause-and-effect relationships. 

## 5. Conclusions

In this paper, we reported significant differences in the plasma concentrations of peptide hormones between obese and lean adult Emiratis. Despite the small number of participants, a notable relationship between the circulating fasting GLP-1 levels and obesity and IR was observed. Restoring the disordered peptide hormones balance in obesity by targeting nutrient sensors in selective regions of the gut or by combined administration of peptide hormone mimetics represent a primary potential therapeutic target to improve the prevention and management of obesity and its comorbidities. Understanding peptide hormones biology is the first step in this regard. To this end, we follow up with the obese individuals after they have undergone bariatric surgery, investigate changes in their peptide hormones profile and include analyses on gut microbiota composition.

## Figures and Tables

**Table 1:** Comparison of demographic, anthropometric, and biochemical parameters between lean and obese participants.

	Lean (Mean ± SD or *n* (%))	Obese (Mean ± SD or *n* (%))	*p*-Value
Age (years)	29.67 ± 10.73	29.95 ± 9.13	0.565
Female	19 (61.3)	27 (62.8)	0.896
BMI (kg/m^2^)	22.49 ± 1.93	43.12 ± 6.83	<0.001 *
WC (cm)	76.70 ± 14.83	123.29 ± 17.76	<0.001 *
Appropriate	23 (74.2)	0 (0)	<0.001 *
Increased risk	8 (25.8)	43 (100)	
WtHR	0.48 ± 0.05	0.74 ± 0.08	<0.001 *
Normal	16 (51.6)	0 (0)	<0.001 *
High	15 (48.4)	43 (100)	
PBF (%)	26.62 ± 9.39	46.13 ± 30	<0.001 *
Adequate	24 (77.4)	0 (0)	<0.001 *
Increased risk	7 (22.6)	41 (95.3)	
FBS (mg/dL)	91.67 ± 11.03	103.11 ± 27.29	0.003 *
Insulin (mU/mL)	3.71 ± 4.41	22.0 ± 14.01	<0.001 *
HOMA-IR	0.86 ± 1.03	5.65 ± 4.14	<0.001 *
Leptin (ng/mL)	20.94 ± 7.96	55.18 ± 18.58	<0.001 *
GLP-1 (pM)	12.64 ± 8.57	23.99 ± 12.83	<0.001 *
GLP-2 (ng/mL)	2.29 ± 1.06	3.59 ± 1.76	<0.001 *
CCK (pg/mL)	68.64 ± 34.39	53.01 ± 28.16	0.037 *
PYY (pg/mL)	61.85 ± 33.99	74.98 ± 50.51	0.355
Ghrelin (pg/mL)	796.08 ± 266.79	531.75 ± 96.12	<0.001 *

* Denotes statistical significance (*p* < 0.05) between groups. Independent-sample *t*-test was used for normally distributed continuous variables (age, BMI, WC, WtHR, PBF, insulin, leptin, GLP-1, GLP-2, CCK), Mann–Whitney U-test for skewed continuous variables (PYY, ghrelin, FBS, and HOMA-IR), and Chi-square test was used for categorical variables. BMI, body mass index; WC, waist circumference; PBF, percentage body fat; WtHR, waist-to-height ratio; FBS, fasting blood sugar; HOMA-IR, Homeostatic Model Assessment of Insulin Resistance; peptide hormones: GLP-1 and GLP-2, glucagon-like peptides 1 and 2; CCK, cholecystokinin; PYY, peptide YY.

**Table 2:** Correlations between peptide hormones and markers of obesity and IR.

		GLP-1	GLP-2	Insulin	Leptin	CCK	PYY	Ghrelin
WC (M)	*r*	0.687 ^a^	0.423 ^a^	0.713 ^a^	0.801 ^a^	0.266 ^a^	0.21 ^b^	−0.722 ^b^
	*p*	<0.001 *	0.025 *	<0.001 *	<0.001 *	0.172	0.28	<0.001 *
WC (F)	*r*	0.382 ^a^	0.286 ^a^	0.61 ^a^	0.762 ^a^	−0.346 ^a^	0.07 ^b^	−0.603 ^b^
	*p*	0.01 *	0.057	<0.001 *	<0.001 *	0.020 *	0.65	<0.001 *
PBF (M)	*r*	0.644 ^a^	0.517 ^a^	0.655 ^a^	0.854 ^a^	−0.143 ^a^	0.26 ^b^	−0.681 ^b^
	*p*	<0.001 *	0.007 *	<0.001 *	<0.001 *	0.484	0.2	<0.001 *
PBF (F)	*r*	0.224 ^a^	0.211 ^a^	0.525 ^a^	0.714 ^a^	−0.384 ^a^	0.07 ^b^	−0.593 ^b^
	*p*	0.139	0.164	<0.001 *	<0.001 *	0.009 *	0.66	<0.001 *
BMI	*r*	0.426 ^a^	0.403 ^a^	0.614 ^a^	0.801 ^a^	−0.356 ^a^	0.09 ^b^	−0.680 ^b^
	*p*	<0.001 *	<0.001 *	<0.001 *	<0.001 *	0.002 *	0.44	<0.001 *
WtHR	*r*	0.549 ^a^	0.444 ^a^	0.651 ^a^	0.756 ^a^	−0.291 ^a^	0.18 ^b^	−0.706 ^b^
	*p*	<0.001 *	<0.001 *	<0.001 *	<0.001 *	0.012 *	0.13	<0.001 *
Insulin	*r*	0.699 ^a^	0.418 ^a^	-	0.474 ^a^	−0.064 ^a^	0.11 ^b^	−0.495 ^b^
	*p*	<0.001 *	<0.001 *	-	<0.001 *	0.589	0.34	<0.001 *
FBS	*r*	0.242 ^b^	0.175 ^b^	0.29 ^b^	0.278 ^b^	−0.193 ^b^	0.17 ^b^	−0.309 ^b^
	*p*	0.039 *	0.139	0.013 *	0.017 *	0.102	0.14	0.008 *
HOMA-IR	*r*	0.738 ^b^	0.368 ^b^	0.992 ^b^	0.588 ^b^	−0.071 ^b^	0.14 ^b^	−0.514 ^b^
	*p*	<0.001 *	0.001 *	<0.001 *	<0.001 *	0.553	0.25	<0.001 *

Spearman correlation was conducted for correlations involving FBS, HOMA-IR, PYY, and ghrelin, while Pearson correlation was conducted for correlations involving other variables: ^a^ Pearson correlation, ^b^ Spearman correlation, *r* = correlation coefficient. * Statistically significant correlation (*p* < 0.05). BMI, body mass index; WC, waist circumference; PBF, percentage body fat; WtHR, waist-to-height ratio; FBS, fasting blood sugar; HOMA-IR, Homeostatic Model Assessment of Insulin Resistance; peptide hormones: GLP-1 and GLP-2, glucagon-like peptides 1 and 2; CCK, cholecystokinin; PYY, peptide YY; M, males; F, females.

**Table 3:** Partial correlations, adjusting for BMI, between peptide hormones and markers of obesity and IR.

		GLP-1	GLP-2	Insulin	Leptin	CCK	PYY	Ghrelin
BMI ^	*r*	0.426 ^a^	0.403 ^a^	0.61 ^a^	0.801 ^a^	−0.356 ^a^	0.093 ^b^	−0.680 ^b^
	*p*	<0.001 *	<0.001 *	<0.001 *	<0.001 *	0.002 *	0.435	<0.001 *
WC (M)	*r*	0.361 ^a^	−0.224 ^a^	0.324 ^a^	0.095 ^a^	0.099 ^a^	0.060 ^b^	−0.146 ^b^
	*p*	0.064	0.260	0.099	0.636	0.622	0.766	0.466
WC (F)	*r*	0.312 ^a^	0.015 ^a^	0.297 ^a^	0.150 ^a^	0.012 ^a^	0.130 ^b^	−0.154 ^b^
	*p*	0.039 *	0.923	0.050	0.330	0.936	0.400	0.317
PBF (M)	*r*	0.281 ^a^	−0.058 ^a^	0.110 ^a^	0.245 ^a^	0.346 ^a^	0.154 ^b^	0.103 ^b^
	*p*	0.174	0.783	0.601	0.238	0.091	0.464	0.625
PBF (F)	*r*	−0.044 ^a^	−0.163 ^a^	0.070 ^a^	−0.053 ^a^	−0.071 ^a^	0.164 ^b^	−0.040 ^b^
	*p*	0.776	0.290	0.652	0.732	0.648	0.287	0.796
WtHR	*r*	0.447 ^a^	0.203 ^a^	0.275 ^a^	0.071 ^a^	0.103 ^a^	0.209 ^b^	−0.302 ^b^
	*p*	<0.001 *	0.087	0.019 *	0.553	0.389	0.078	0.019 *
Insulin	*r*	0.612 ^a^	0.236 ^a^	-	−0.038 ^a^	0.209 ^a^	0.070 ^b^	−0.093 ^b^
	*p*	<0.001 *	0.046 *	-	0.749	0.077	0.557	0.438
FBS	*r*	0.074 ^b^	0.027 ^b^	0.032 ^b^	−0.161 ^b^	−0.046 ^b^	0.149 ^b^	−0.044 ^b^
	*p*	0.535	0.825	0.787	0.176	0.704	0.213	0.716
HOMA	*r*	0.667 ^b^	0.170 ^b^	0.987 ^b^	0.009 ^b^	0.273 ^b^	0.100 ^b^	−0.094 ^b^
	*p*	<0.001 *	0.154	<0.001 *	0.940	0.021 *	0.405	0.432

^ Correlation was presented without adjustment for BMI. Spearman partial correlation was conducted for correlations involving FBS, HOMA-IR, PYY, and ghrelin, while Pearson partial correlation was performed for correlations involving other variables. ^a^ Pearson partial correlation. ^b^ Spearman partial correlation. * Statistically significant correlation (*p* < 0.05). BMI, body mass index; WC, waist circumference; PBF, percentage body fat; WtHR, waist-to-height ratio; FBS, fasting blood sugar; HOMA-IR, Homeostatic Model Assessment of Insulin Resistance; GLP-1 and GLP-2, glucagon-like peptides 1 and 2; CCK, cholecystokinin; PYY, peptide YY; M, males; F, females.

**Table 4:** Predictors of markers of obesity and IR.

	Predictors	OR	*p*-Value	R Square	95% CI
HOMA-IR ^a^	BMI GLP-1	1.229 1.221	<0.001 0.002	0.764	1.108–1.363 1.079–1.382
Weight status ^b^	Leptin GLP-1	1.367 1.239	0.002 0.033	0.898	1.117–1.672 1.018–1.508
WC ^c^	BMI GLP-1	3.876 1.196	0.027 0.048	0.871	1.169–12.848 1.001–1.430
WtHR ^d^	BMI GLP-1	10.276 1.591	0.023 0.021	0.900	1.373–76.934 1.072–2.363
PBF ^e^	BMI GLP-1	4.847 0.717	0.008 0.020	0.910	1.503–15.636 0.542–0.949

^a^ Reference category: HOMA-IR < 1.8 vs. HOMA-IR ≥ 1.8. Variables entered in the model: GLP-1, leptin, ghrelin, and BMI. ^b^ Reference category: lean vs. obese. Variables entered in the model: leptin, GLP-1, and ghrelin. ^c^ Reference category: appropriate WC vs. increased WC. Variables entered in the model: leptin, GLP-1, and BMI. ^d^ Reference category: normal WtHR vs. high WtHR. Variables entered in the model: GLP-1 and BMI. ^e^ Reference category: normal PBF vs. elevated PBF. Variables entered in the model: leptin, GLP-1, and BMI. HOMA-IR, Homeostatic Model Assessment of Insulin Resistance; GLP-1, glucagon-like peptides 1; PBF, percentage body fat; WC, waist circumference; WtHR, waist-to-height ratio.

## Data Availability

The raw data presented in this study are openly available in Mendeley Data repository: https://data.mendeley.com/datasets/w297t54m98/1 (accessed on 7 February 2022) at https://doi.org/10.17632/w297t54m98.1.
